# Senescent B cells regulate CD38 expression via FOXO1 in pneumonia resulting from *PIK3CD* (R437C) mutations

**DOI:** 10.1093/lifemedi/lnaf030

**Published:** 2025-11-25

**Authors:** Ju Liu, Yuxin Bai, Jianing Tang, Peiyao Jin, Yanmei Huang, Lu Yang, Ying Wang, Xiaochuan Wu, Chaohong Liu

**Affiliations:** Department of Pathogen Biology, School of Basic Medicine, Tongji Medical College and State Key Laboratory for Diagnosis and Treatment of Severe Zoonotic Infectious Diseases, Huazhong University of Science and Technology, Wuhan 430030, China; Department of Pathogen Biology, School of Basic Medicine, Tongji Medical College and State Key Laboratory for Diagnosis and Treatment of Severe Zoonotic Infectious Diseases, Huazhong University of Science and Technology, Wuhan 430030, China; Department of Pathogen Biology, School of Basic Medicine, Tongji Medical College and State Key Laboratory for Diagnosis and Treatment of Severe Zoonotic Infectious Diseases, Huazhong University of Science and Technology, Wuhan 430030, China; Department of Immunology, School of Medicine, Yangtze University, Jingzhou 434100, China; Department of Pathogen Biology, School of Basic Medicine, Tongji Medical College and State Key Laboratory for Diagnosis and Treatment of Severe Zoonotic Infectious Diseases, Huazhong University of Science and Technology, Wuhan 430030, China; Department of Pathogen Biology, School of Basic Medicine, Tongji Medical College and State Key Laboratory for Diagnosis and Treatment of Severe Zoonotic Infectious Diseases, Huazhong University of Science and Technology, Wuhan 430030, China; Department of Pediatrics, The Second Xiangya Hospital, Central South University, Changsha 410011, China; Department of Pediatrics, The Second Xiangya Hospital, Central South University, Changsha 410011, China; Department of Pathogen Biology, School of Basic Medicine, Tongji Medical College and State Key Laboratory for Diagnosis and Treatment of Severe Zoonotic Infectious Diseases, Huazhong University of Science and Technology, Wuhan 430030, China

**Keywords:** *PIK3CD* mutation, APDS, B cell, cellular senescence, CD38

## Abstract

Activated phosphoinositide 3-kinase delta syndrome (APDS) is a primary immunodeficiency characterized by hyperactivated lymphocytes and recurrent infections. This study presents a 2.5-year-old patient with a novel *PIK3CD* gene mutation (c.1309C>T; p. R437C) derived from his mother. We explored the immunological consequences of this mutation in both the patient and his mother, revealing defects in T cell differentiation, B cell maturation, and mitochondrial function. Notably, we found that the elevated CD38 expression on B cells is a key factor driving B cell senescence, mitochondrial dysfunction, and increased transitional B cell proportion, contributing to the observed immunodeficiency, such as diminished serum antibodies. Further investigations of the PI3K/AKT/mTOR pathway highlight a preferential activation of mTORC2 over mTORC1. We also demonstrate that the transcription factor FOXO1, a downstream molecule of PI3K/AKT signaling, regulates CD38 expression by binding to the promotor of the *CD38* gene, linking this pathway to B cell dysfunction. This novel mutation expands the spectrum of *PIK3CD* mutations associated with APDS and provides new insights into the molecular mechanisms underlying B-cell senescence and other immune dysregulation. Moreover, targeting the AKT–FOXO1 axis could offer therapeutic potential to reverse B-cell dysfunction and improve immune responses in patients with *PIK3CD* mutations.

## Introduction

Class I phosphoinositide 3-kinase (PI3K) plays a pivotal role in cellular metabolism and immunological function [1]. Comprising a catalytic subunit p110 and a regulatory subunit p85 (most prevalent), PI3K maintains a basal activity state through the interaction between the C2 domain of its catalytic subunit and the N-SH2 domain of its regulatory subunit [2]. This inhibitory association can be relieved by phosphorylated tyrosine [3], thereby enabling Class I PI3K to catalyze the conversion of phosphatidylinositol (4,5)-bisphosphate (PI (4,5) P2) into phosphatidylinositol (3,4,5)-bisphosphate (PI (3,4,5) P3). Extensive human research and murine experiments have demonstrated that an excessively activated state of PI3K often leads to malignancies and dysregulation within the immune system [4, 5].

By utilizing next-generation sequencing, various sites of PI3K mutations have been identified, with particular emphasis on gain-of-function (GOF) mutations associated with activated phosphoinositide 3-kinase delta syndrome (APDS) [6]. APDS includes APDS1 and APDS2. APDS1 is attributed to mutations in the *PIK3CD* gene on human chromosome 1, which encodes the p110δ subunit; APDS2 is attributed to the *PIK3R1* gene, which encodes the p85α subunit [7]. p110δ is one of the 3 catalytic subunit variants in Class I PI3K. While other variants, p110α and p110β are ubiquitously expressed in all cell sets, p110δ is predominantly expressed in immune cells [8]. The most frequently observed APDS mutations are APDS1 c.3061G>A (p.E1021K) mutation and APDS2 c.1425 + 1G> (A, C, T) (p.434–475del) mutation [6]. Patients carrying those mutations as well as other APDS mutations often present a range of symptoms associated with hyperactivation and proliferation of lymphocytes, such as hepatosplenomegaly, lymphadenopathy, and autoimmune diseases; however, they also manifest immunodeficiency-related symptoms, including recurrent respiratory infections, reduced levels of immunoglobulins, and developmental delay [6].

Further investigations of patients with APDS have revealed immune dysregulation. In B cell populations, most cases exhibit reduced total CD19^+^ B cell counts and impaired antibody class switching, accompanied by increased proportions of transitional B cells and plasma cells [9]. Serum levels of IgG and IgA are significantly decreased, while serum IgM levels are elevated [10]. However, the underlying mechanism responsible for abnormal B cell differentiation processes and changes in B cell activation states remains unclear. In T cell populations, a decrease in the number of CD3^+^ T cells has been observed along with an elevated level of T cell senescence, leading to peripheral T cell exhaustion [11, 12]. Some cases also display reduced naïve CD4^+^ T cells, increased effector CD8^+^ T cells, and an altered ratio of CD4^+^ to CD8^+^ T cells [6, 13]. However, there are significant variations in immunophenotypic features among patients.

In this article, we present two cases of APDS patients harboring a novel missense mutation (c.1309C>T; p. R437C) in the *PIK3CD* gene from a family, a child with his mother. The child exhibits recurrent lung infections, cutaneous red papules, hepatic insufficiency, and coagulation dysfunction. By exploring the proportional and functional changes in the patient and his mother, a heterozygous carrier of this mutant gene, our investigation reveals several key findings on immune system dysregulation. In our cases, disorders including abnormal T-cell differentiation, impaired B-cell maturation, over-activated B cells and PI3K/AKT/mTOR signaling pathway, aberrant T/B-cell metabolism, and increased T/B-cell senescence can be observed. Paradoxical to some previous reports, our results demonstrate reductions in plasma cells and serum Ig isotypes, IgM and IgG. The decreases and disordered subpopulations in T cells and B cells are similar to previous reports. Notably, there is a marked increase in transitional B cells, and the expression level of CD38 rises ubiquitously among B-cell subpopulations. By exploring at the molecular level, this study aims to contribute to the understanding of how this specific GOF *PIK3CD* mutation leads to immune dysregulation with a focus on peripheral T cells and B cells.

## Results

### Clinical features and genetic findings in the patient with primary immunodeficiency

**Figure 1.**
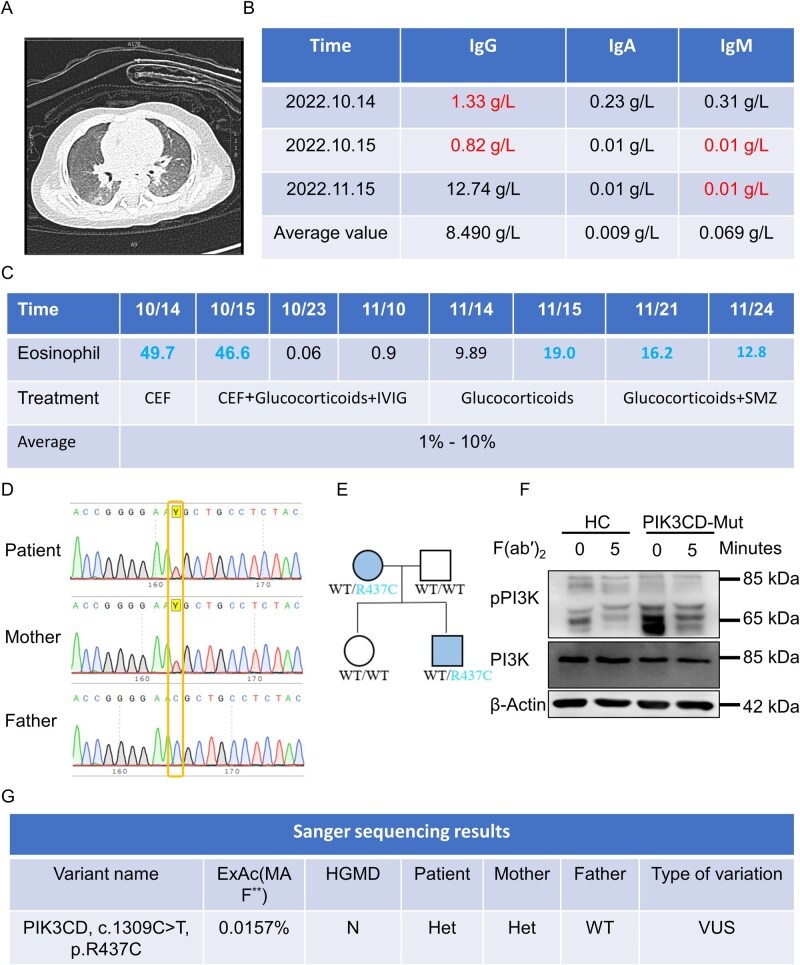
Identification of a *de novo PIK3CD* variant in a patient with early-onset immune dysregulation disease. (A) CT of a severely infected lung in a child who received the first dose of DPT vaccine after birth. (B) Serum immunoglobulin (IgM/IgG/IgA) levels during hospitalization. (C) Partial treatment strategies and eosinophilic levels in child during hospitalization. (D) Genetic test report of the affected child and his family members. He and his mother are heterozygous carriers of *PIK3CD* c.1309C>T (p.R437C) mutation. (E) Pedigrees of the families with heterozygous in *PIK3CD* c.1309C>T (p.R437C) mutation. (F) Protein levels of PI3K and activation levels at rest and under stimulation with Biotin-F(ab′)_2_ anti-human Ig(M+G) antigen in patient and HC PBMCs. pPI3K, phosphorylated PI3K. (G) Report of genetic testing by Sanger sequencing of the peripheral blood of the affected children. MAF, minor allele frequency; ExAC, MAF derived from the human exon database; HGMD, human gene mutation database. Y for included and N for not included. Het, heterozygous; WT, wild-type; VUS, variant of uncertain significance.

The proband was a 2.5-year-old boy who was admitted to the hospital 3 months after birth due to cough, dyspnea, and fever following the administration of the first-dose diphtheria tetanus pertussis (DPT) vaccine. Physical examination revealed scattered, needle-like erythematous papules protruding from the skin, along with dry and desquamated areas on the trunk. Bilateral coarse breath sounds were auscultated without rales. Chest X-ray showed blurred lung textures with patchy, high-density fuzzy shadows in both lungs, and chest CT revealed scattered pulmonary exudates (Fig. 1A). Fiberoptic bronchoscopy identified chondromalacia in the epiglottis and inflammation in the bronchial epithelium. Those image findings supported pneumonia, which was further confirmed to be caused by *Pneumocystis carinii* and *Mycoplasma pneumoniae* by microbiological examination.

Although the patient does not have allergies, pediatric asthma, or any other conditions that could cause eosinophilia, peripheral blood analysis revealed a significant increase in the proportion of eosinophils among the white blood cells, with a peak value of 49.7% (normal range: 1%–10%), suggesting a potential immune disorder instead [14]. Routine treatment with glucocorticoids and intravenous immunoglobulin was initiated to alleviate the inflammation associated with eosinophilia. Meanwhile, antibiotics like cephalosporin and sulfamethoxazole were intermittently applied to the patient during his stay at the hospital (Fig. 1C). Another prominent feature of the patient’s immunological profile was the depletion of serum immunoglobulins. Both IgG and IgM levels were significantly reduced, while IgA levels remained normal, which contradicted previous reports linking *PIK3CD* mutations with increased IgM secretion and decreased IgA levels [10] (Fig. 1B). These immunological findings above indicated significant immune disorders in the patient. Overall, the patient was diagnosed with primary immunodeficiency, severe pneumonia leading to respiratory failure, *Pneumocystis carinii* infection, eosinophilia, hydrocephalus, liver dysfunction, coagulation disorder, and atrial septal defect.

The patient was born to non-consanguineous, healthy parents and has an 8-year-old healthy sister. During the later stage of pregnancy, the mother experienced thrombocytopenia and was treated with leucogen. Notably, the patient has an older sister who passed away at the age of one due to *Pneumocystis carinii* infection. Given the patient’s immunodeficiency and family history, whole-exome sequencing (WES) was performed. WES identified a heterozygous variant of uncertain significance (VUS), c.1309C>T (p.R437C), in the *PIK3CD* gene (NM_005026.5), which is associated with autosomal dominant immunodeficiency type 14A (MIM : 615513). Sanger sequencing confirmed this mutation in both the proband and his mother (Fig. 1D). The results revealed that the patient’s mother was a carrier of the mutant allele in a heterozygous state, while the father exhibited homozygous wild-type for this locus (Fig. 1E).

In conclusion, the identified c.1309C>T (p.R437C) missense mutation in the *PIK3CD* gene on chromosome 1 was inherited from the mother and is a likely contributing factor to the patient’s immunodeficiency. According to the ExAC database, the minor allele frequency of this variant is low in the general population, and it is not listed in the HGMD database. In the Clinvar database, the types of variation were categorized into five: pathogenic, likely pathogenic, VUS, likely benign, and benign. However, due to the uncertain clinical significance of this variant and its lack of clear association with the patient’s immunodeficiency, it is currently classified as a VUS (Fig. 1G). Further investigation is necessary to clarify its potential pathogenic role.

We examined the activation status of PI3K under both resting and antigen-stimulated conditions. The results revealed that, compared to normal cells, patient-derived mutant cells exhibited significantly higher levels of phosphorylated PI3K (pPI3K) even at the resting state (Fig. 1F), indicating aberrant PI3K activation. Following antigen stimulation, pPI3K levels remained elevated in mutant cells compared to controls, though the difference was less pronounced than in the resting condition. Notably, despite reduced total PI3K protein expression in mutant cells, these cells displayed constitutive PI3K activation in the absence of external stimulation. These findings suggest that the PIK3CD mutation leads to decreased PI3K protein levels while simultaneously inducing its constitutive activation under basal conditions.

### *PIK3CD* mutation disrupts T-cell differentiation and immune homeostasis

**Figure 2.**
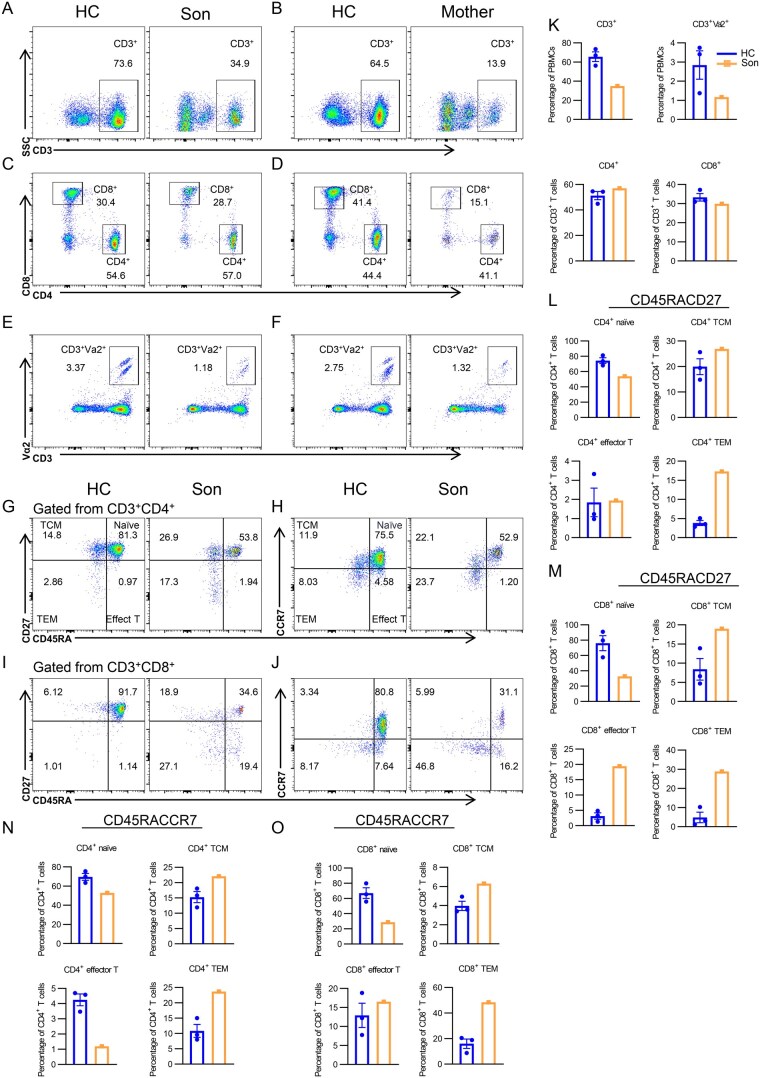
*PIK3CD* mutation disrupts T-cell differentiation and immune homeostasis.(A–J) Flow cytometry analysis of CD3^+^ (A and B), CD4^+^ and CD8^+^ (C and D) T cells, CD3^+^Vα2^+^ (E and F) T cells, naïve (CD45RA^+^CCR7^+^), TCM (CD45RA^−^CCR7^+^), TEM (CD45RA^−^CCR7^−^), effector (CD45RA^+^CCR7^−^) T cells in PBMCs from CD4^+^ (G and H) and CD8^+^ (I and J) in PBMCs from three HCs and the *PIK3CD* mutant patients. Shown are representative dot plots. (K–O) Statistics of percentage (±SEM) of CD3^+^, CD4^+^, CD8^+^, and CD3^+^Vα2^+^ T cells (K–M), naïve (CD45RA^+^CCR7^+^), TCM (CD45RA^−^CCR7^+^), TEM(CD45RA^−^CCR7^−^), effector (CD45RA^+^CCR7^−^) T cells in CD3^+^CD4^+^ (L) and CD3^+^CD8^+^ (M) T cells from HCs and the son with *PIK3CD* mutant (HC = 3). (N–O) Statistics of percentage (±SEM) of naïve (CD45RA^+^CCR7^+^), TCM (CD45RA^−^CCR7^+^), TEM (CD45RA^−^CCR7^−^), effector (CD45RA^+^CCR7^−^) T cells in CD3^+^CD4^+^ (N), and CD3^+^CD8^+^ (O) T cells from three HCs and the mother with *PIK3CD* mutant.

We used the peripheral blood mononuclear cells (PBMCs) from both the patient and his mother to investigate the impact of *PIK3CD* mutations on T-lymphocyte differentiation by flow cytometry (Fig. S1A). Remarkably, our patient exhibited a significant reduction of peripheral CD3^+^ T cell proportion compared to the healthy control (HC) (Fig. 2A and 2K). Within the CD3^+^ T cells, the patient exhibited a significant decrease of CD3^+^Vα2^+^ T cells, while no substantial differences were found in CD4^+^ and CD8^+^ T cells (Fig. 2C, 2E, and 2K). The mother exhibited a similar peripheral T-cell scenery, with total CD3^+^ T cells, CD8^+^ T cells, and CD3^+^Vα2^+^ T cells decreased, while CD4^+^ T cells also showed a slight decrease (Fig. 2B, 2D, 2F, and Fig. S1D). CD3^+^Vα2^+^ T cells represent a group of T cells with a specific segment of the variable region of TCR α-chain that can recognize MHC molecules with specific antigens. Thus, the reduced proportion of CD3^+^Vα2^+^ T cells may suggest that the T cells of this particular TCR subset are continuously activated and depleted, leading to a decline in number.

Further analysis of CD4^+^ T cell subsets, the naïve T cells, central memory T cells (TCM), effector memory T cells (TEM), and effector T cells, revealed a dysregulated T-cell differentiation process. Two detecting strategies were applied to separate the subsets of T cells, based on the expression of CD27 and CD45RA, and the expression of CCR7 and CD45RA, respectively. The two strategies demonstrated similar results: compared to HC, the proportions of naïve T cells were reduced, and TEM and TCM were elevated among both CD4^+^ and CD8^+^ T cells in the patient. The proportion of CD8^+^ effector T cells was increased, while the proportion of CD4^+^ effector T cells showed a decrease in the patient, potentially correlating with the infection type of the patient (Fig. 2G–J, 2L–O). On the contrary, the mother showed an elevated CD4^+^ effector T cell proportion and a decrease in CD8^+^ effector T cell proportion compared to HC. Other T cell subsets of the mother exhibited similar characteristics to the son, with the trends of TEM and TCM rising, and naïve T cells reducing (Fig. S1B–J). The slight difference between the results of the two different gating strategies could possibly be attributed to the marker’s feature. CCR7 is a chemokine receptor that reflects the migratory ability of T cells, especially homing to lymphoid organs; thus, it can be downregulated in inflammatory or infectious states. CD27 expression is closely associated with T cell activation and differentiation and reflects less about migration ability than CCR7.

In conclusion, the exhaustion of peripheral CD3^+^ T cells (especially CD8^+^ T cells and CD3^+^Vα2^+^ T cells) can be observed in this *PIK3CD* mutant patient and his mother. Additionally, both the patient and his mother exhibited an aberrant T-cell differentiation state, suggesting that PIK3CD mutation disrupts T-cell differentiation and immune homeostasis. The variants of T-cell subsets between the patient and his mother may be attributed to the existence of infection and age-related immunological changes.

### *PIK3CD* mutation impairs B-cell maturation and influences CD38 expression

**Figure 3.**
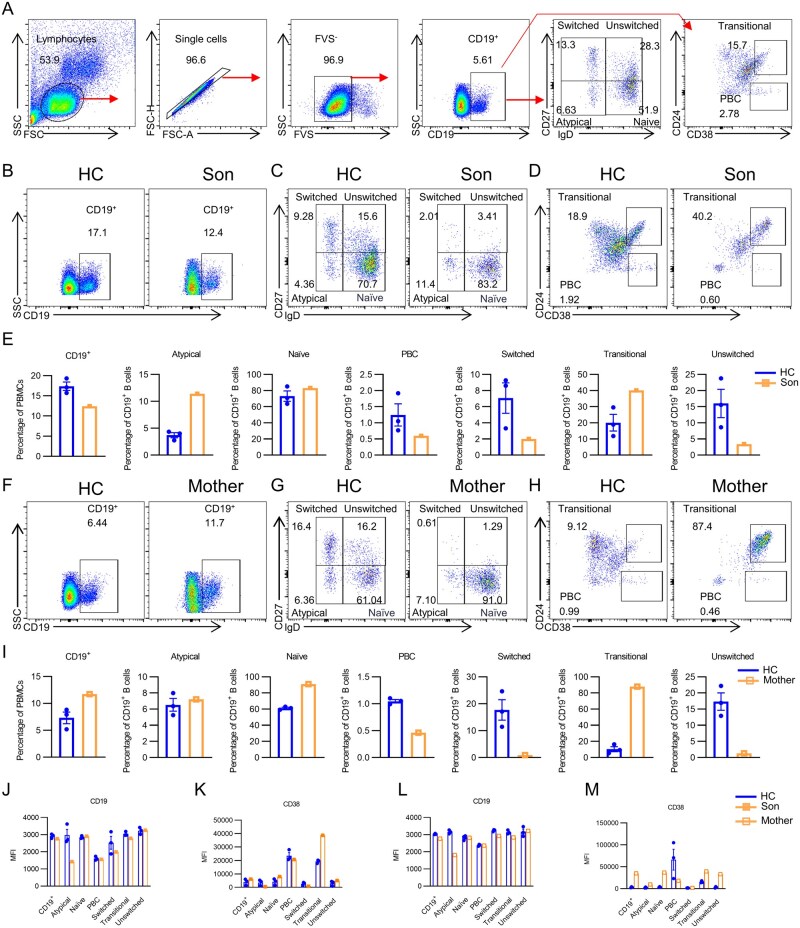
PIK3CD mutation impairs B-cell maturation and influences CD38 expression.(A) Flowchart of gate strategy for B cell subpopulation analysis. (B–D, F–H) Flow cytometry analysis of B-cell subtypes for CD19^+^, naïve, memory, transitional, and plasmablast cells in PBMCs from HCs and patients with *PIK3CD* mutations. The analysis includes age-matched child HCs and the child patient (B–D), as well as age-matched adult HCs and the mother (F–H). Representative dot plots are shown for each comparison. (E and I) Statistics of the percentage (±SEM) of CD19^+^ naïve B, memory B, transitional B, and plasmablast cells from three HCs and the patient with *PIK3CD* mutation. Data are presented for age-matched child HCs and the child patient (E), and for age-matched adult HCs and the mother (I) (HC = 3). (J–M) Analysis of the MFI of CD19 and CD38 in B-cell subtypes of PBMCs from HCs and patients with *PIK3CD* mutant. (HC = 3)

Previous studies reported that GOF *PIK3CD* mutation causes a decrease in B-lymphocytes and diminished antibody secretion [9, 15]. Thus, we further investigated the B-cell populations to evaluate the effect of *PIK3CD* mutation on B cells (Fig. 3A). The flow cytometry results of the patient demonstrated a significant decrease in the proportion of CD19^+^ B cells within PBMCs in comparison to HC (Fig. 3B and 3E). We separated atypical B cells, naïve B cells, switched memory B cells, and unswitched memory B cells based on the expression of IgD and CD27, and transitional B cells and plasmablast cells (PBC) based on the expression of CD38 and CD24. The results showed that compared to HC, there were elevated proportions of transitional B cells and naïve B cells alongside decreased proportions of PBC, switched memory B cells, and unswitched memory B cells in the patient’s peripheral blood (Fig. 3C–E). The above results indicated impaired B-cell maturation, as the young-staged B cells accumulated and antibody-secreting B cells were insufficient. Furthermore, a rise in atypical B cell proportion can be observed in the patient (Fig. 3E). Atypical B cells are unable to effectively produce antibodies and are usually overrepresented in chronic infections or autoimmune diseases, indicating an exhausted state derived from memory B cells. Given the patient’s pulmonary infection and his primary immunodeficiency, we deduce that the insufficient effector B cells were unable to support effective anti-pathogen effects, ultimately leading to memory cell exhaustion.

The mother’s peripheral B-cell populations were also detected. Different from the son, the mother exhibited an elevation in the proportion of CD19^+^ B cells among PBMC in comparison to HC, and only a slight elevation can be seen in atypical B cells (Fig. 3F and 3I). Other changes demonstrated similar trends to the son, that the proportions of transitional B cells and naïve B cells were increased, and the proportions of PBC, switched memory B cells, and unswitched memory B cells were depleted (Fig. 3G–I). Those alterations indicated shared features among the carriers of the *PIK3CD* mutant gene.

Pivotal functional molecules in B cells also exhibited altered expression levels. We examined the expression levels of CD19 and CD38 molecules as indicated by the mean fluorescence intensity within each B cell subset. In our patient, while in total CD19^+^ B cells, naïve B cells, PBC, and unswitched memory B cells, the expression of CD19 was at the same level as HC, reduced expression levels of CD19 can be observed across atypical B cells, switched memory B cells, and transitional B cells (especially within atypical B cells) (Fig. 3J). The CD19 expression trends were consistent in the mother’s B cells, also showing a down-regulated trend, especially for atypical B cells (Fig. 3L). As a signal transduction molecule expressed throughout all stages of B-cell development (from the pro-cellular stage to the plasma cell stage), CD19 helps B-cell signal transduction and is associated with the development and maturation processes [16]. Thus, the decreased levels of CD19 indicated damages in B-cell activities, possibly contributing to the maturation and differentiation disorders.

The expression of CD38 seemed to be the opposite of CD19 levels, with the expression levels of CD38, increased across total CD19^+^ B cells, naïve B cells, unswitched memory B cells, and transitional B cells, but decreased in PBC (for both the son and the mother) (Fig. 3K and 3M). As a marker molecule, CD38 expression levels vary at different developmental stages of B cells [17]. CD38 is expressed on early-stage and B cells in bone marrow and spleen, not on mature B cells; while on activated plasma cells, CD38 re-expresses at a high level [18]. Additionally, CD38 promotes the proliferation and differentiation of transitional II B cells in the spleen, and the process requires the involvement of various signaling pathways like PI3K, Lyn, Fyn, Btk, and Erk [19]. Thus, the elevated expression levels of CD38 correlated with *PIK3CD* GOF mutation in this case, which may add to explaining the increased peripheral transitional B-cell populations.

These findings collectively indicate a severe disruption in B-cell maturation following PIK3CD mutation in this case. The detection of CD19 and CD38 expression levels further reflects the pathogenesis of the disordered immune system and indicates potential functional changes.

### *PIK3CD* mutation leads to over-activated B cells and PI3K/AKT/mTOR signaling pathways with FOXO1 binding to *CD38* promotor

**Figure 4.**
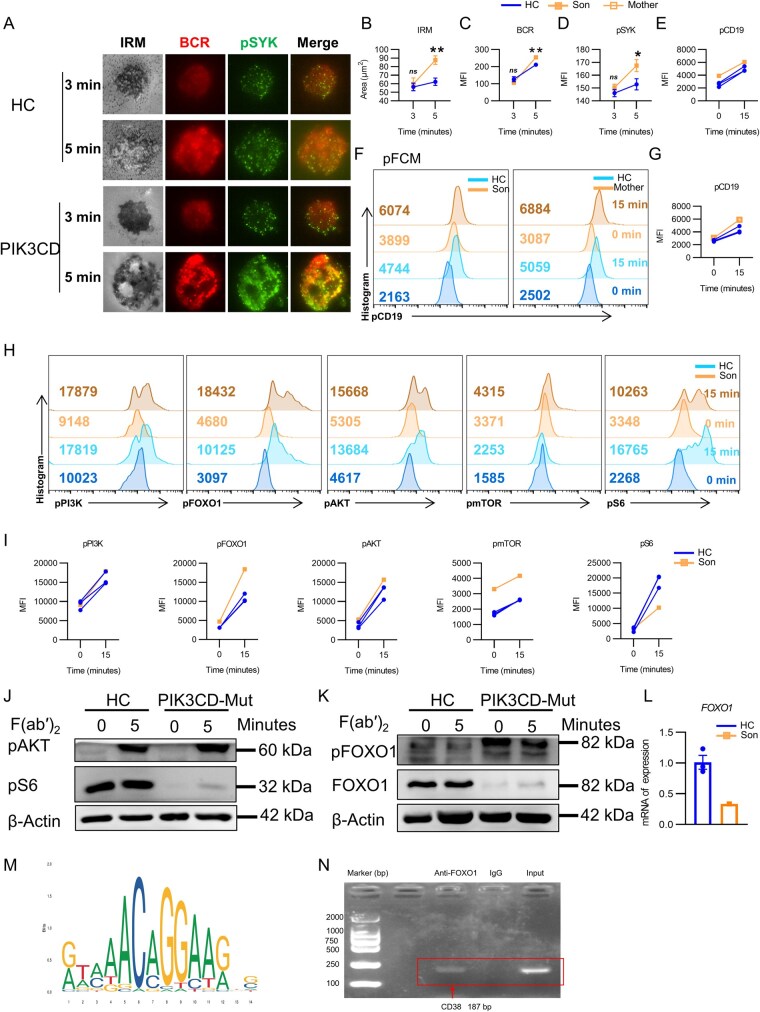
PIK3CD mutation leads to over-activated B cells and PI3K/AKT/mTOR signaling pathways with FOXO1 binding to *CD38* promotor. (A) Sorted B cells from HCs and *PIK3CD* mutant patient were incubated with 10 µg/mL AF546–Biotin-Fab′–anti-Ig tethered to lipid bilayers at 37°C for 3 and 5 min, then fixed, permeabilized, and stained with anti-pSYK, followed by Alexa Fluor 488 goat anti-rabbit IgG. Cells were analyzed by Tirf microscope. BCR, B cell Receptor; IRM, Interference Reflection Microscopy; pSYK, phosphorylated spleen tyrosine kinase. Shown are representative images captured using Nikon TIRFm. (B–D) The B-cell area in the contact zone (B), the MFI of BCR (C), and the MFI of pSYK (D) in the contact zone from HCs and *PIK3CD* mutant patients were quantified using NIS-­Elements AR 5.0.1 software. Statistical evaluation was performed using the two-tailed Student’s *t*-test. (E–I) PBMCs from HCs and the *PIK3CD* mutant patients were pre-incubated with anti-CD19, followed by stimulation with Biotin-F(ab′)_2_ anti-human Ig(M+G) antigen for 0 and 15 min. After fixation and permeabilization, cells were stained with anti-pCD19, anti-pPI3K, anti-pFOXO1, anti-pAKT, anti-­pmTOR, and anti-pS6 and analyzed by flow cytometry. The MFI of pCD19, pPI3K, pFOXO1, pAKT, pmTOR, and pS6 in CD19^+^ B cells was quantified by FlowJo 10 software. Representative histograms were shown (F and G) (HC = 3). (J and K) WB analysis of the protein levels of pAKT and pS6, pFOXO1 and FOXO1 at rest and under stimulation with Biotin-F(ab′)_2_ anti-human Ig(M+G) antigen in patient and HC PBMCs. (L) RT-PCR analysis *FOXO1* mRNA levels after *PIK3CD* mutation. (M) JASPAR website predicted the motif sequence of *FOXO1* binding to the *CD38* promoter. (N) ChIP-PCR analysis of the *FOXO1* binding with the *CD38* motif region in human PBMCs by using the *CD38* promoter primer. The band is 187 bp.

Given the previous evidence of *PIK3CD* mutations affecting B-cell activation [10], we isolated peripheral B cells from the patient and his mother to assess the role of PI3K signaling in B-cell activation. Interference Reflection Microscopy was utilized to visualize the changes in B-cell morphology, BCR clusters, and phosphorylated SYK (pSYK) signalosome at different time points (3 and 5 min) following Biotin-F(ab′)_2_ anti-human Ig(M + G) antigen stimulation, which activates B-cells and allows for the detection of related changes. After 5 min of stimulation, a significant expansion of B cells was observed under the microscope in both *PIK3CD* mutant group and the HC group (Fig. 4A). Upon antigenic stimulation, BCRs usually aggregate into ­clusters and form immune synapses, followed by the phosphorylation of SYK, a vital downstream kinase in the BCR signaling pathway. At 3 min, BCR clustering and initial pSYK activation were observed, indicating the early stages of B cell activation. At 5 min, stronger clustering of BCR and increased pSYK fluorescence can be seen, suggesting amplified signaling and receptor engagement (Fig. 4A). Meanwhile, compared to the HC group, B cells in *PIK3CD* mutant group exhibited an over-activated state as indicated by the line-connected scatter plots. In the patient, the contact area between the B cells and the antigen-presenting surface increased more significantly from 3 to 5 min, and the contact area of the patient cells was significantly larger than that of the HC cells (*P *< 0.01) (Fig. 4B). Similarly, the MFI of BCR clusters and pSYK levels increase from 3 to 5 min and were both significantly higher in the patient cells compared to HC (*P *< 0.05, *P *< 0.01, respectively) (Fig. 4C and 4D). The above results highlighted a clear dysregulation in B cell responses caused by the mutation, particularly affecting receptor activation and downstream signaling.

Phospho-flow cytometry (pFCM) was then performed to assess the phosphorylation levels of CD19 (pCD19) in B cells from the son and the mother compared to HC. At baseline (0 min), both the son and mother exhibited slightly elevated pCD19 compared to HC, indicating potential pre-activation. Following stimulation, the son’s and mother’s B cells showed significantly higher MFI of pCD19 compared to HC, as evidenced by the pronounced rightward shift in the histograms and quantitative analysis of MFI (Fig. 4E–G). These findings demonstrated that the mutation enhanced CD19 phosphorylation, suggesting hyperactivation of the CD19 signaling pathway in response to stimulation.

We then investigated the phosphorylation of several important signaling molecules associated with PI3K signaling pathway, including pPI3K, pmTOR, pAKT, pS6, and pFOXO1 in B cells upon stimulation. The results showed that both the patient and HC cells showed an increase in pPI3K levels after 15 min of stimulation (Fig. 4H and 4I). The patient cells exhibited a significantly higher level of pmTOR at both 0 min and 15 min compared to HC cells (Fig. 4H and 4I). As a key regulator of cell growth and metabolism, higher pmTOR levels indicate an enhanced metabolic state in mutant cells. Additionally, both the patient and HC cells showed an increase in pAKT and pS6 levels after 15 min (Fig. 4I). The Western blot results also ­demonstrated that, upon stimulation, pS6 levels were lower in ­PI3KCD-mutant patient than in HCs, whereas pAKT levels were slightly higher, consistent with the pFCM findings (Fig. 4J and 4K). The mother’s B-cell signaling was also detected (Fig. S2), and the results were similar to the son’s. These findings correlated with previous cases, suggesting that *PIK3CD* mutations lead to enhanced PI3K/AKT/mTOR signaling [20], with individual-specific nuances that could influence clinical presentation. Further investigation is warranted to delineate the functional implications of these changes. mTOR binds to different proteins to form two functionally distinct complexes, mTORC1 and mTORC2. In the PI3K signaling pathway, pS6 is a downstream molecule of mTORC1, while pAKT is a downstream molecule of mTORC2. Hence, our findings suggested that this novel mutation predominantly affects mTORC2 activation, with minimal impact on mTORC1. Both our Western blot and pFCM results show that mutant cells were associated with enhanced pAKT activation but diminished pS6 activation relative to HCs (Fig. 4I and 4J), but previous studies do not seem to have examined mTORC1 and mTORC2 in such detail [7]. In Gulbu Uzel’s article, the authors examined the level of pS6 in the basal state, but there was a large individual variation in the level of pS6 in patients, and the level of pS6 was elevated after stimulation with anti-CD3 [12]. In our study, the mutant cells were stimulated with biotinylated F(ab′)_2_ anti-human Ig(M + G) antigen, and the level of pS6 was found to be significantly reduced in the mutant cells by both pFCM and Western blot assays. This is different from previous findings, and it is possible that this is also related to the differences in the mutation sites.

FOXO1 is an important transcription factor that usually induces cell apoptosis and block cell cycle progression [21]. Along with the activation of the PI3K/AKT signaling pathway, the downstream effector pFOXO1 exhibited a substantially larger increase in B cells from patient compared to HC. However, the level of total FOXO1 protein was significantly reduced (Fig. 4K). We also examined the mRNA level of *FOXO1* in the mutant cells by RT-PCR. As we suspected, the *PIK3CD* mutation also affected the gene expression of FOXO1 (Fig. 4L). Thus, we hypothesize that the mechanism underlying CD38 overexpression in PIK3CD-mutant cells may be caused by the transcription factor FOXO1, which may directly bind to the *CD38* promoter to inhibit the expression of the CD38 molecule.

To investigate, motif sequence prediction and chromatin immunoprecipitation (ChIP) were performed. Generated using the JASPAR database, the predicted motif sequence for FOXO1 binding to the *CD38* promoter (NC_000004.12) is illustrated. The position weight matrix shows the nucleotide preferences at each position of the binding site, with a clear consensus sequence indicating the specific recognition motif (Fig. 4M). This prediction suggests that FOXO1 has a high-affinity binding site in the *CD38* promoter region, supporting its role as a transcriptional regulator. The ChIP-PCR analysis provides direct evidence of FOXO1 binding to the predicted motif region within the CD38 promoter in PBMCs. Using a primer specific to the *CD38* promoter region, the “Anti-FOXO1” lane shows a strong PCR product compared to the IgG control, confirming specific enrichment. The “Input” lane serves as a control for total chromatin availability, validating the experimental results (Fig. 4N).

These findings collectively demonstrate that FOXO1 binds directly to the promoter of *CD38* and negatively regulates its expression. This provides critical insights into the regulatory mechanism of FOXO1 in transcriptional control of CD38, with the potential to become a therapeutic target to regulate B-cell activities.

### *PIK3CD* mutation impairs mitochondrial function

Among the patient’s and his mother’s B-cell subsets, the expression of CD38 molecules exhibited a broad rise. A previous article showed that CD38 interacts with the PI3K/AKT/mTOR signaling pathway to regulate metabolic reprogramming, reducing NAD^+^ levels [22], thereby impairing mitochondrial function and efficiency. Hence, we next detected the metabolic states among T cells and B cells.

**Figure 5.**
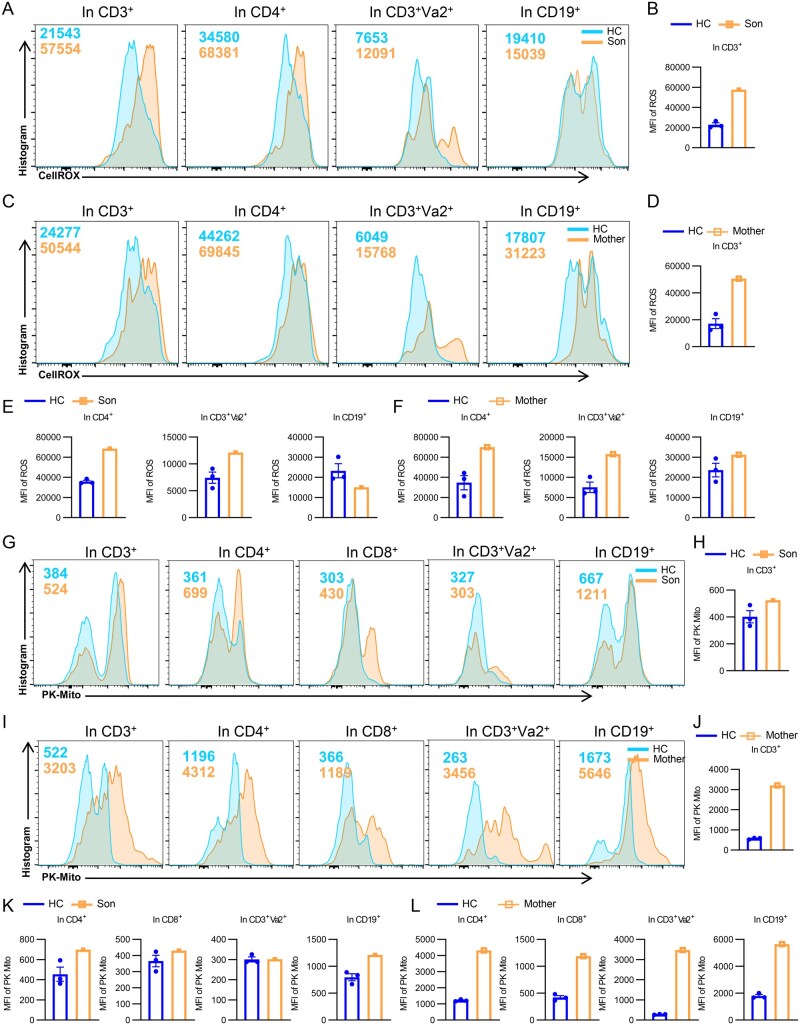
PIK3CD mutation impairs mitochondrial function. (A, C) Flow cytometry analysis of CellROX in T and B cell subsets of PBMCs from HCs and the patients with *PIK3CD* mutant. Shown are representative histogram. (B, D–F) Analysis of the MFI of ROS in CD3^+^, CD4^+^, CD3^+^Vα2^+^ T cells, and CD19^+^ B cells of PBMCs from HCs and the patients with *PIK3CD* mutant (HC = 3). (G and I) Flow cytometry analysis of PK-Mito in T and B cell subsets of PBMCs from HCs and the patients with *PIK3CD* mutant. Shown are representative histogram. (H, J–L) Analysis of the MFI of PK-Mito in CD3^+^, CD4^+^, CD3^+^Vα2^+^ T cells, and CD19^+^ B cells of PBMCs from HCs and the patients with *PIK3CD* mutant (HC = 3).

We first assessed the reactive oxygen species (ROS) production of lymphocytes from the patient, the mother, and HCs. ROS is mainly generated by the mitochondrial respiratory chain and NADPH oxidase during metabolic activities. High levels of ROS usually originate from overactive or impaired mitochondrial electron transport chains, suggesting oxidative stress or metabolic diseases [23]. For the patient, significantly elevated ROS levels were observed across total CD3^+^ T cells, CD4^+^ T cells, and CD3^+^Vα2^+^ T cells as indicated by the MFI of CellROX compared to HC (Fig. 5A, 5B, and 5E). However, for CD19^+^ B cells, the son exhibited a decreased ROS level (Fig. 5E). For the mother, increased ROS production can be observed among all detected cell subsets (Fig. 5C, 5D, and 5F). These findings suggested that *PIK3CD* mutation in both the son and the mother leads to dysregulated ROS production across multiple lymphocyte subsets. The elevated ROS levels, particularly in T cells, indicate a hyperactive metabolic and oxidative state that could contribute to immune cell dysfunction and inflammation.

Additionally, PK-Mito levels were analyzed in T and B lymphocytes from the patient, the mother, and HCs. As a mitochondrial probe, PK-Mito levels reflect the efficiency of pyruvate, a product of glycolysis, to enter the mitochondria, thus revealing the activity of mitochondrial oxidative metabolism and energy-consuming state [24]. In CD3^+^ T cell, CD4^+^ T cell, CD8^+^ T cell, and CD19^+^ B cell populations, the patient exhibited significantly higher PK-Mito levels compared to HC, as evidenced by the rightward shift in histograms and increased MFI values (Fig. 5G, 5H, and 5K). In CD3^+^Vα2^+^ T cells, the PK-Mito level was about the same between the patient and HC, showing no evident elevation in the patient (Fig. 5K). These results indicate an overall hyperactive mitochondrial metabolic state in the patient’s lymphocytes, with particularly pronounced effects in B cells and CD4^+^ T cells. The enhanced mitochondrial metabolism was even more significant among the mother’s lymphocytes, as all measured cell subsets exhibited substantially higher levels of PK-Mito (Fig. 5I, 5J, and 5L), reflecting significantly dysregulated mitochondrial functions.

In conclusion, the above results elucidated the impact of *PIK3CD* mutations on mitochondrial functions and cellular oxidative stress, presenting the links between *PIK3CD* mutation, increased CD38 expression, and impaired mitochondrial function.

### *PIK3CD* mutation causes increased senescence within T cells and B cells

**Figure 6.**
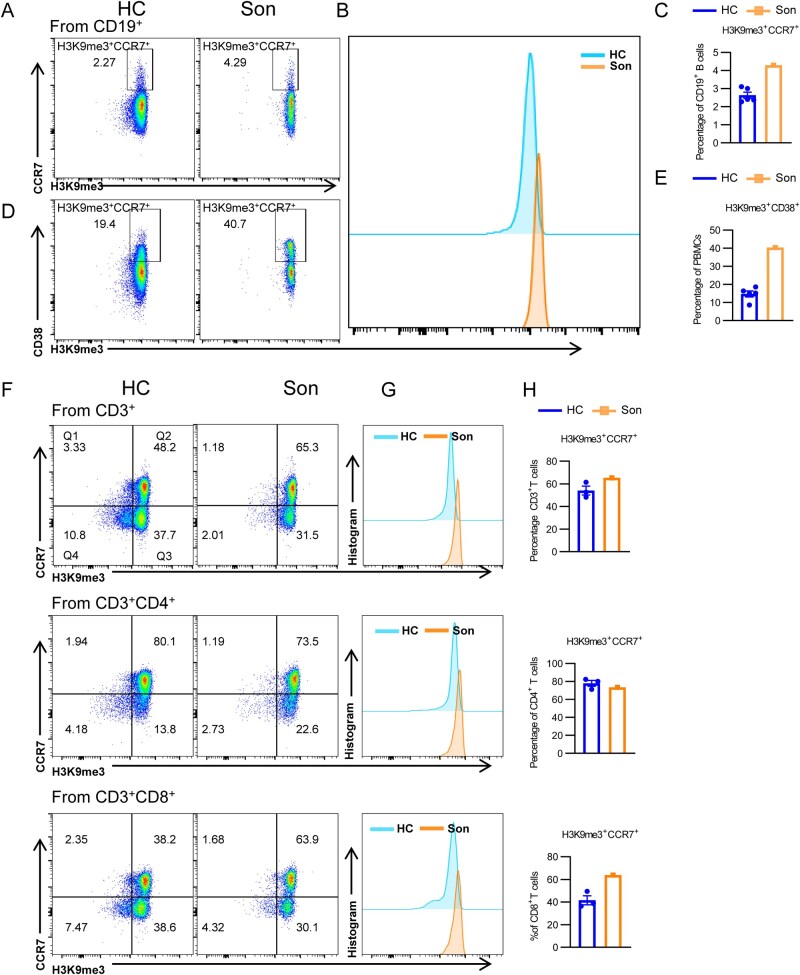
PIK3CD mutation causes increased senescence within T cells and B cells.(A–C) Flow cytometry analysis of senescent B cells (H3K9me3^+^CCR7^+^) of PBMCs from HCs and the son with *PIK3CD* mutant. Flow cytometry analysis of senescent B cells (H3K9me3^+^CCR7^+^) in PBMCs from HCs and the son with the *PIK3CD* mutation. The representative histogram of senescent B cells is shown in (B), and the percentage (±SEM) of senescent B cells is analyzed in (C) (HC = 5). (D and E) Flow cytometry analysis of H3K9me3^+^CD38^+^ B cells of PBMCs from HCs and the son with *PIK3CD* mutant. The representative histogram is shown in (D), and the percentage (±SEM) of senescent B cells is analyzed in (E) (HC = 5). (F–H) Flow cytometry analysis of senescent T cells (H3K9me3^+^CCR7^+^) of PBMCs from HCs and the son with *PIK3CD* mutant. The representative flow diagrams of H3K9me3^+^CCR7^+^ T cells in CD3^+^, CD3^+^CD4^+^, and CD3^+^CD8^+^ T cells are shown in (F). The representative histograms of H3K9me3^+^CCR7^+^ T cells in CD3^+^, CD3^+^CD4^+^, and CD3^+^CD8^+^ T cells are shown in (G), and the percentages (±SEM) of H3K9me3^+^CCR7^+^ T cells in CD3^+^, CD3^+^CD4^+^, and CD3^+^CD8^+^ T cells are analyzed in (H) (HC = 3).

Cellular senescence is a state of irreversible cell cycle arrest triggered by various factors, characterized by a prolonged and generally irreversible halt in cell-cycle, accompanied by secretory features, macromolecular damage, and altered metabolism. The senescence-associated secretory phenotype (SASP) produced by senescent cells contributes to harmful side effects [25, 26]. The expression of CD38 demonstrates age-related differences and suggests a potential role in promoting cellular senescence [27]. Therefore, we further investigated the cellular senescence state by detecting the intracellular level of the histone H3 lysine-9 methylation (H3K9me3), a key epigenetic modification marker of heterochromatin [28]. Senescence-associated heterochromatin foci (SAHF) increase during cellular senescence and may also be protective in response to oxidative stress and chronic inflammation/infection. Thus, H3K9me3 can preliminarily reflect the amount of the senescence state of the cell [29]. According to the flow cytometry results, in CD19^+^ B cells, the percentage of H3K9me3^+^CCR7^+^ cell was significantly higher in the patient compared to HC (Fig. 6A–C). Similarly, the proportion of H3K9me3^+^CD38^+^ cells was markedly elevated among PBMCs in the patient (Fig. 6D and 6E). In T cells, a higher percentage of H3K9me3^+^CCR7^+^ cell was also observed in total CD3^+^ T cell populations in the patient compared to HC (Fig. 6F–H). Further investigations on CD4^+^ and CD8^+^ T-cell subsets revealed that the elevated senescence level of T cells was mainly attributed to CD8^+^ T cells, for the H3K9me3^+^CCR7^+^ cells showed an increase among CD8^+^ T cells. For CD4^+^ T cells, the senescence marker H3K9me3 showed no significant variance between the mutant patient and HC (Fig. 6H). These overall results demonstrate that PIK3CD mutation leads to an accumulation of senescent T and B lymphocytes, which additionally suggests a link between the *PIK3CD* mutation and immune dysfunction. As B-cell senescence increased, the antibody-producing efficiency would be weakened, likely contributing to diminished serum IgG and IgM and impaired adaptive immune responses.

## Discussion

In this study, we report two cases of a novel mutation in the *PIK3CD* gene, identified in a boy proband and his mother, both are heterozygous carriers. The mutation, c.1309C>T (p.R437C), represents a previously unreported GOF mutation associated with APDS. Our findings expand the spectrum of reported *PIK3CD* mutations globally. Among previously reported cases of APDS caused by *PI3K* mutations, the most common variants are c.3061G>A (p.E1021K) in the *PIK3CD* gene and c.1425 + 1G> (A, C, T) (p.434–475del) in the *PIK3R1* gene [30]. Patients with APDS typically present with common features such as hyperactivated lymphocytes, immunodeficiency, recurrent infections, developmental delay, and hepatosplenomegaly. Immunologically, these cases are characterized by T/B-cell differentiation defects, markedly increased transitional B cells, and reduced antibody secretion (except for IgM). In our case, the boy proband exhibited recurrent lung infections, cutaneous red papules, hepatic insufficiency, and coagulation dysfunction, indicative of immunodeficiency. Interestingly, his mother, despite carrying the same mutation, did not exhibit such clinical symptoms. This phenomenon may stem from age-related differences in immune metabolism. Although the mother exhibits no clinical symptoms, the significant elevation in both PK-mito and ROS suggests that immune cells compensate for the functional defects caused by the gene mutation by enhancing mitochondrial oxidative metabolism. Immunological analysis of peripheral blood from both individuals revealed abnormalities consistent with prior reports, including T-cell differentiation defects, B-cell maturation impairments, and markedly reduced serum immunoglobulins of IgG and IgM. Furthermore, we conducted in-depth explorations into the PI3K/AKT/mTOR signaling pathway, B-cell activation, mitochondrial function, and cellular senescence in T and B cells.

Similar to other *PIK3CD* mutations, this novel variant leads to hyperactivation of the PI3K/AKT/mTOR signaling pathway. However, our findings suggest that this mutation predominantly affects mTORC2 activation, with minimal impact on mTORC1. Specifically, upon antigen stimulation, patient B cells displayed significantly elevated pAKT levels, whereas pS6 levels increased at a slower rate and lower than HCs after 15 min of stimulation, highlighting preferential activation of mTORC2 (the downstream molecule of AKT) over mTORC1 (the downstream molecule of S6). However, further investigation is required to determine whether other PIK3CD mutations differentially affect mTORC1 or mTORC2 activation.

A striking finding in this study was the pervasive overexpression of CD38 on patient B-cell populations (except for PBCs). Previous research in murine models has shown that CD38 ­promotes the differentiation of transitional II B cells [19]. This observation provides a potential explanation for the significant increase in transitional B cells observed in APDS patients and identifies CD38 as a potential therapeutic target for reversing B cell differentiation abnormalities in future clinical applications.

Elevated CD38 expression may also account for the mitochondrial dysfunction and increased oxidative stress observed in patient T and B cells. Studies in cervical cancer cells have demonstrated that CD38 overexpression reduces NAD^+^ levels [22], thereby impairing mitochondrial function and efficiency. This aligns with our findings, which showed increased mitochondrial ROS and dysfunction in the patient’s immune cells. Additionally, CD38 overexpression may promote cellular senescence[27], consistent with our observation of significantly elevated levels of the senescence marker H3K9me3 in the patient’s T and B cells. The rising senescent B cells contribute to impaired antibody production. Hence, the depletion of immunoglobulin may be attributed to both increased B-cell senescence and impaired B-cell maturation. More importantly, the mechanism underlying CD38 overexpression in *PIK3CD-*mutant cells involves the transcription factor FOXO1, a downstream effector of PI3K/AKT signaling. Our findings indicate that FOXO1 directly associates with the promoter of CD38 in healthy PBMCs. In mutant cells, the reduction of FOXO1 expression coincided with increased CD38 expression, supporting the conclusion that FOXO1 acts as a transcriptional repressor to regulate the expression of CD38.

One unique and severe clinical feature in the proband was peripheral eosinophilia, which necessitated regular immunoglobulin therapy to maintain normal eosinophil levels. Eosinophils are typically confined to the bone marrow, and only very few amounts of eosinophils exist in the peripheral blood [31]. Interestingly, PI3K signaling has been shown to promote eosinophil adhesion and migration [32]. We speculate that the hyperactive PI3K signaling in this patient facilitates eosinophil migration into the peripheral blood. However, given the short half-life of eosinophils (6–12 h), elucidating the precise impact of *PIK3CD* mutations on eosinophil trafficking remains a significant challenge for our future research.

**Figure 7.**
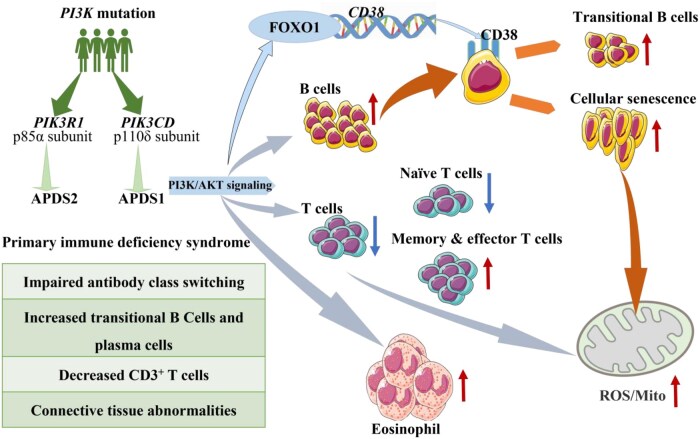
Pathogenic mechanism of a novel *PIK3CD* mutation in activated PI3K-Delta syndrome. Mutations in *PIK3R1* or *PIK3CD* lead to hyperactivation of the PI3K/AKT signaling pathway, causing the primary immunodeficiency syndrome APDS. This study identifies a novel *PIK3CD* mutation that results in distinct immune dysregulation phenotypes. Hyperactive PI3K/AKT signaling suppresses the transcription factor FOXO1, leading to the upregulation of its target gene, *CD38*, on B cells. Elevated CD38 expression drives B cells toward a senescent state, characterized by mitochondrial dysfunction and increased ROS production. This process impairs B-cell maturation, resulting in an accumulation of transitional B cells and diminished antibody class switching. Concurrently, the mutation alters T cell homeostasis, causing a decrease in naïve T cells and an increase in memory/effector T cells, alongside a rise in eosinophil counts. These findings link the PI3K/AKT/FOXO1 axis directly to B cell senescence and immunodeficiency in APDS, highlighting potential therapeutic targets.

In summary, we report a novel *PIK3CD* mutation in this article and establish a link between PI3K signaling and B cell senescence for the first time. Mechanistically, our findings suggest that CD38 expression is enhanced by the PI3K–AKT–FOXO1 axis, leading to B-cell senescence, mitochondrial dysfunction, and transitional B-cell elevation (Fig. 7). Based on this pathway, we propose that inhibitors targeting AKT or FOXO1 could potentially reverse B cell senescence, thereby restoring antibody production and possibly ameliorating immunodeficiency in patients with *PIK3CD* mutations.

## Research limitations

While this study elucidates the impact of the *PIK3CD* R437C mutation on T and B lymphocyte development and further clarifies the mechanisms underlying the senescent phenotype of B cells, several limitations should be acknowledged. First, our investigation is based on only two related individuals from a single family. This small sample size restricts our ability to fully assess the broader impact of this specific mutation on the immune system and precludes validation at a population level. Second, although we observed a significant elevation in ­neutrophil counts in the pediatric patient, we were unable to further investigate the functional alterations or the underlying cause of this neutrophilia. Finally, the absence of a corresponding animal model prevented a systemic, *in vivo* evaluation of the long-term effects of this mutation on immune cell dynamics and disease-related phenotypes. Therefore, future research incorporating larger patient cohorts and appropriate animal models is essential to more comprehensively and systematically unravel the mechanisms of immune dysregulation driven by *PI3K* mutations and to fully understand their clinical implications.

## Methods

### Research ethics

A Chinese family containing two patients with a *de novo PIK3CD* mutation (c.1309C>T; p. R437C) was enrolled in this study. Age-matched HC subjects (children and adults) were recruited from unrelated families and underwent thorough health assessments. All control subjects were free of immune disorders. Written informed consents, approved by the ethics committee of Tongji Medical College, Huazhong University of Science and Technology (Ethical approval number: 2025S070), were obtained from the parents of all participants.

### Isolation of PBMCs

For human PBMC extraction, blood from peripheral circulation was collected and centrifuged at 3000 rpm for 10 min, 2 mL of serum was frozen and stored in a −80 freezer. The rest of the sample was mixed with PBS and added to the centrifuge tube containing Ficoll-Hypaque solution, centrifuged with a gradual acceleration to 2000 rpm and a slow deceleration at the end.

### Whole genome sequencing

The genomic DNA of the submitted samples was extracted, fragmented, ligated, amplified and purified, and then a DNA library was prepared by hybridization capture method, and then the exonic regions and the paracrine intronic regions (20 bp) of 20,099 genes in the human whole exome were detected by high-throughput sequencing platform. The sequencing data were aligned with the reference sequence of human genome hg19 (GRCh37), the coverage and sequencing quality of the target regions were evaluated. Cloned and amplified exons 9–11 of the *PI3KCD* gene from the pediatric patient and family members, followed by sequencing. The sequencing primers were: forward primer TCTCGGGTGGGGTGCC and reverse primer CAGCATCTCTGGGACCC.

### Flow cytometry and antibodies

For surface staining, isolated PBMCs were stained with antibodies for 30 min, then washed twice. Flow cytometric data were acquired with an Attune NxT (Thermo Fisher) and analyzed with FlowJo 10 software (TreeStar, Ashland, OR). Antibodies for B cells: Percp-Cy5.5-anti-CD19 (302230), FITC-anti-CD19 (302206), PE-anti-CD24 (311106), APC-­anti-CD27 (311106), PB-anti-CD38 (356628), and BV510-anti-IgD (348220). Antibodies for T cells: FITC-anti-CD8 (555366), PE-anti-CD4 (357404), APC-anti-CD4 (551980), APC-anti-CD27 (311106), APC-Cy7-anti-CD3 (344818), PE-Cy7-anti-CCR7 (353226), BV605-anti-CD45RA (304134), and PE-Cy7-anti-Vα2 (331422). Antibodies used were obtained from Biolegend and diluted at a ratio of 1:300 for use. During flow cytometry staining, the frozen cells were stained with viability dyes such as FVS (BD, 564997) or 7-AAD (BD, 559925) to distinguish between live and dead cells.

### Phosphoflow cytometry

PBMCs (1.5 × 10^6^) were prepared into single-cell suspension using pre-warmed 2% 1640 medium, then 100 µL biotin-conjugated F(ab′)_2_ anti-human Ig(M + G) (Jackson, 109066127) was used to incubate with the cells. Next, the cells were stimulated at 37°C for 0 or 15 min, then fixed with PFA and membrane-broken by 2× Cytofix/Perm/Wash Bufferto the cells for 15 min. Cells were washed with Perm/Wash Buffer (BD, 554723) to remove PFA and then stained with anti-pPI3K (4228S), anti-pFOXO1 (9461S), anti-pAKT (4060 L), anti-pS6 (4858S), anti-pmTOR (5536S), and anti-pCD19 for 30 min. Antibodies used were obtained from CST.

### Western blot

PBMCs (2.5 × 10^6^) were stimulated with 5 μg/mL Biotin-F(ab′)_2_ anti-human Ig(M + G) for 30 min and then incubated for 5 min at 37°C and lysed by RIPA buffer. Proteins were separated using 8% SDS-PAGE and then incubated with anti-pPI3K, anti-pFOXO1, anti-FOXO1, anti-pAKT, anti-pS6, anti-pmTOR overnight, then incubated with the secondary antibody at room temperature for 1 h.

### Total internal reflection fluorescence microscope

For TIRFm analysis, prepare a clean chamber, lay liposomes on the bottom of the chamber, and incubate at 37°C for 30 min, discard the excess liposomes, add biotin-conjugated F(ab′)_2_ anti-human Ig (M + G) (mAg) and Streptavidin to the chamber, incubate at 37° for 20 min, and the chamber is ready for use. Then, cells were added to the prepared chamber at 37°C for 3 min and 5 min, followed by fixation and permeabilization with 8% PFA. Cells were stained with anti-pSYK (2710S) and Alexa Fluor 488 goat anti-mouse IgG (Thermo, A11001). A Nikon system was used to image the Tirf samples, and NIS elements AR 5.2 software was used for data analysis.

### ROS and PK mito measurement

PBMCs (5 × 10^5^) from HCs and mutant patients were stained with the dymer CellROX™Green (C10444, Invitrogen) for ROS detection, followed by PE-anti-CD19, APC-cy7-anti-CD3, APC-anti-CD4, and PE-Cy7-anti-Vα2 staining for 15 min. For mitochondrial function analysis, HCs and mutant patients’ PBMCs were stained with PKMITOTM Deep Red (PKMDR-1, GENVIVO), followed by PE-anti-CD19, APC-cy7-anti-CD3, APC-anti-CD4, and PE-Cy7-anti-Vα2 for 15 min. Data were collected by using a flow cytometer and analyzed with FlowJo software.

### ChIP assay

ChIP studies were performed using SimpleChIP Enzymatic Chromatin IP Kit according to the manufacturer’s instructions. PBMCs were crosslinked with 1% formaldehyde at room temperature for 20 min and terminated with 1.375 M of glycine. Cytoplasmic lysate and nuclear lysate were added sequentially, and the supernatant was collected for sonication to fragment the crosslinked chromatin into 100 to 1000 bp fragments. Solubilized chromatin was immunoprecipitated with an anti-FOXO1 antibody, and PCR amplification was performed on a qTOWER3/G PCR instrument. The primers for the binding site of FOXO1 were as follows: forward, TGCCAGTCAGAATGGTGATCATT; reverse, CCCAGTAATGAGATTGCTGGGTC.

### Statistical analysis

The normality of the data was assessed, and statistical analysis was performed using a two-tailed unpaired Student’s *t*-test (GraphPad Prism 9.5). Flow cytometric data were acquired with an Attune NxT (Thermo Fisher) and analyzed with FlowJo 10 software (TreeStar, Ashland, OR). Error bars indicate the mean ± SEM, and differences were considered statistically significant at *P *< 0.05.

## Supplementary Material

lnaf030_Supplementary_Data

## Data Availability

The data sets and any other raw data that support the findings of this study are available from the corresponding author upon reasonable request.
