# Supplementary material for: Senescent B cells regulate CD38 expression via FOXO1 in pneumonia resulting from PIK3CD (R437C) mutations
Source: Life Med. 2025 Nov 25;4(5):lnaf030. doi: 10.1093/lifemedi/lnaf030 (PMC12853000; doi:10.1093/lifemedi/lnaf030)
Supplement: lnaf030_Supplementary_Data [file lnaf030_supplementary_data.zip › Fig_PIK3CD mutant Revised_0828_figure_SI.pdf]

Senescent B Cells Regulate CD38 Expression via FOXO1 in Pneumonia Resulting from *PIK3CD* (R437C) Mutations

Ju Liu[1], Yuxin Bai[1], Jianing Tang[1], Peiyao Jin[3], Yanmei Huang[1], Lu Yang[1], Ying Wang[2,*], Xiaochuan Wu[2,*], Chaohong Liu[1,*]

[1]Department of Pathogen Biology, School of Basic Medicine, Tongji Medical College and State Key Laboratory for Diagnosis and Treatment of Severe Zoonotic Infectious Diseases, Huazhong University of Science and Technology, Wuhan 430030, China

[2]Department of Pediatrics, The Second Xiangya Hospital, Central South University, Changsha 410011, China

[3]Department of Immunology, School of Medicine, Yangtze University, Jingzhou 434100, China

Correspondence: chaohongliu80@126.com (C.L.), xiaochuanwu@csu.edu.cn (X.W.), wangying001@csu.edu.cn (Y.W.)

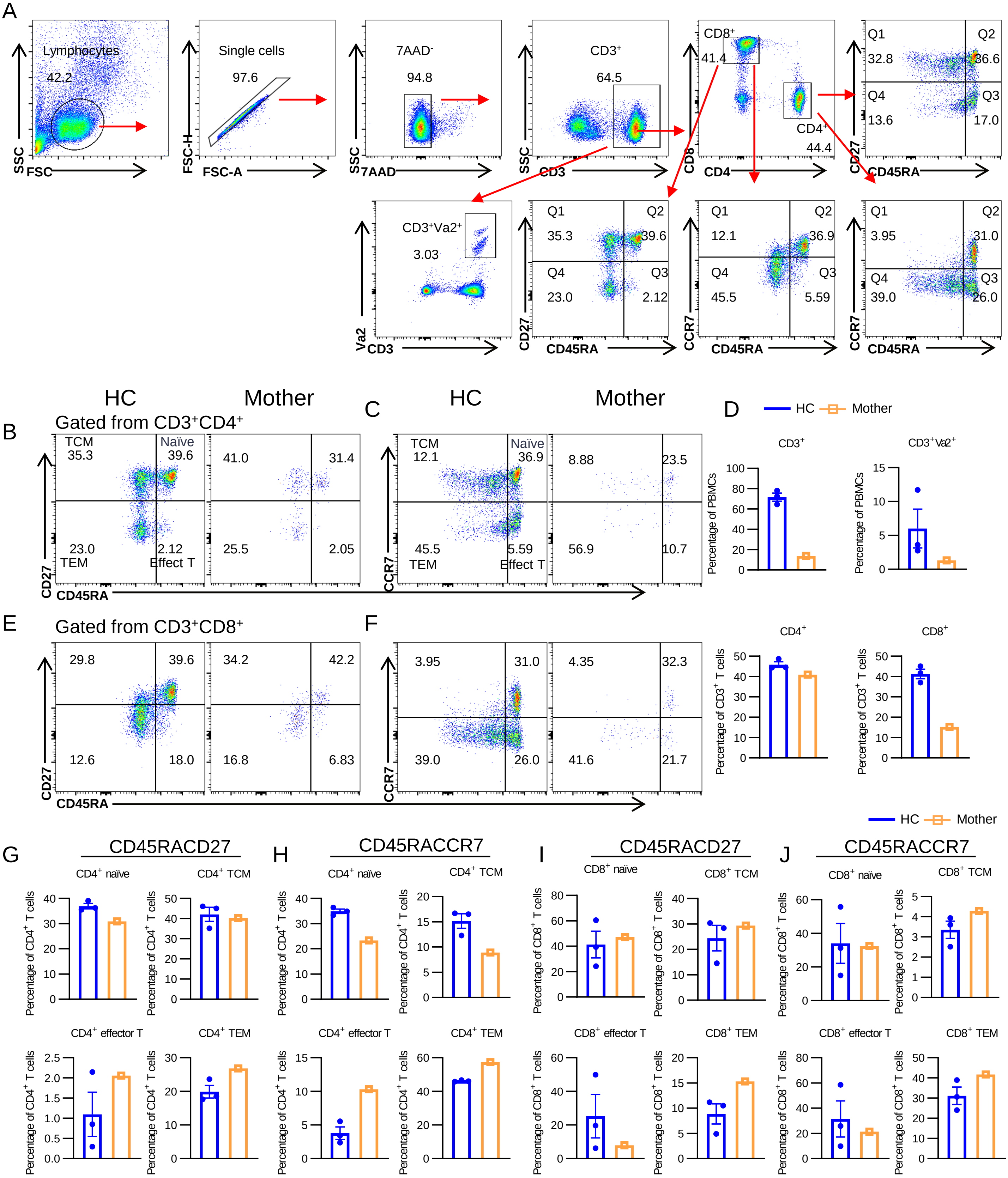

A
Lymphocytes
42.2
Single cells
97.6
7AAD-
94.8
CD3+
64.5
CD8+
41.4
CD4+
44.4
Q1 32.8
Q2 36.6
Q4 13.6
Q3 17.0
CD3+Va2+
3.03
Q1 35.3
Q2 39.6
Q4 23.0
Q3 2.12
Q1 12.1
Q2 36.9
Q4 45.5
Q3 5.59
Q1 3.95
Q2 31.0
Q4 39.0
Q3 26.0
B
C
HC
Mother
Gated from CD3+CD4+
TCM 35.3
Naïve 39.6
23.0 TEM
2.12 Effect T
41.0
31.4
25.5
2.05
TCM 12.1
Naïve 36.9
45.5 TEM
5.59 Effect T
8.88
23.5
56.9
10.7
D
HC
Mother
CD3+
CD3+Va2+
Percentage of PBMCs
E
F
Gated from CD3+CD8+
29.8
39.6
12.6
18.0
34.2
42.2
16.8
6.83
3.95
31.0
39.0
26.0
4.35
32.3
41.6
21.7
CD4+
CD8+
Percentage of CD3+ T cells
G
H
I
J
CD45RACD27
CD45RACCR7
CD4+ naïve
CD4+ TCM
CD4+ effector T
CD4+ TEM
CD8+ naïve
CD8+ TCM
CD8+ effector T
CD8+ TEM
Percentage of CD4+ T cells
Percentage of CD8+ T cells
SSC
FSC
FSC-H
FSC-A
7AAD
CD3
CD8
CD4
CD27
CD45RA
Va2
CCR7

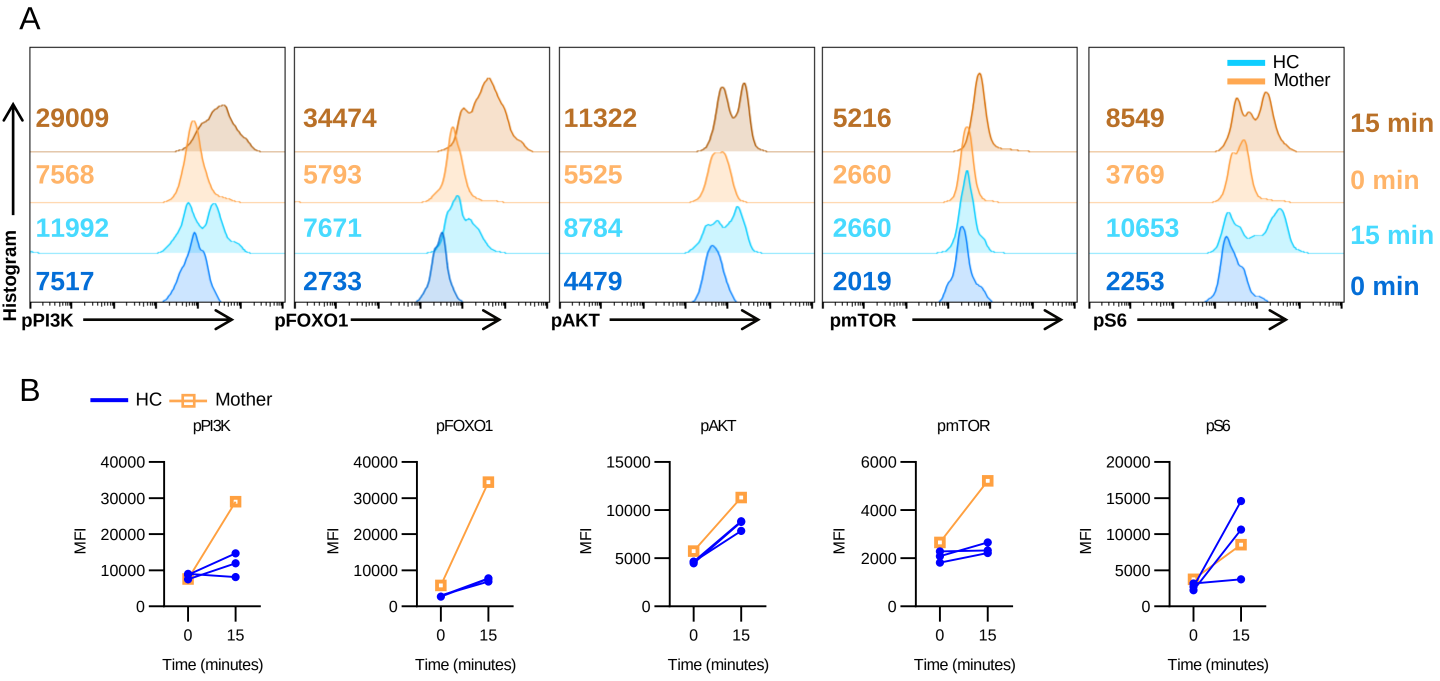
A
HC
Mother
29009
7568
11992
7517
34474
5793
7671
2733
11322
5525
8784
4479
5216
2660
2660
2019
8549
3769
10653
2253
15 min
0 min
15 min
0 min
Histogram
pPI3K
pFOXO1
pAKT
pmTOR
pS6
B
HC
Mother
pPI3K
pFOXO1
pAKT
pmTOR
pS6
MFI
Time (minutes)
