# Supplementary material for: Senescent B cells regulate CD38 expression via FOXO1 in pneumonia resulting from PIK3CD (R437C) mutations
Source: Life Med. 2025 Nov 25;4(5):lnaf030. doi: 10.1093/lifemedi/lnaf030 (PMC12853000; doi:10.1093/lifemedi/lnaf030)
Supplement: lnaf030_Supplementary_Data [file lnaf030_supplementary_data.zip › Senescent B Cells Regulate CD38 Expression via FOXO1 in Pneumonia Resulting from PIK3CD_SI.docx]

**Senescent B Cells Regulate CD38 Expression via FOXO1 in** **Pneumonia Resulting from *PIK3CD* (R437C) Mutations**

Ju Liu^1^, Yuxin Bai^1^, Jianing Tang^1^, Peiyao Jin^3^, Yanmei Huang^1^, Lu Yang^1^, Ying Wang^2,*^，Xiaochuan Wu^2,*^, Chaohong Liu^1,*^

^1^Department of Pathogen Biology, School of Basic Medicine, Tongji Medical College and State Key Laboratory for Diagnosis and Treatment of Severe Zoonotic Infectious Diseases, Huazhong University of Science and Technology, Wuhan 430030, China

^2^Department of Pediatrics, The Second Xiangya Hospital, Central South University, Changsha 410011, China

^3^Department of Immunology, School of Medicine, Yangtze University, Jingzhou 434100, China

Correspondence: [chaohongliu80@126.com](mailto:chaohongliu80@126.com) (C.L.), xiaochuanwu@csu.edu.cn (X.W.), [wangying001@csu.edu.cn](mailto:wangying001@csu.edu.cn) (Y.W.)

**Figure legends**

**Figure S1. *PIK3CD* mutation disrupts T-cell differentiation and immune homeostasis.**

(A) Flowchart of gate strategy for T cell subpopulation analysis. (B, C) Flow cytometry analysis of naïve (CD45RA^+^CD27^+^), TCM (CD45RA^−^CD27^+^), TEM(CD45RA^−^CD27^−^), effector (CD45RA^+^CD27^−^) T cells in PBMCs from CD3^+^CD4^+^ (B) and CD3^+^CD8^+^ (C) T cells of the mother with *PIK3CD* mutant. Shown are representative dot plots. (D) Statistics of percentage (±SEM) of CD3^+^, CD4^+^, CD8^+^ and CD3^+^Vα2^+^ T cells from HCs and the mother with *PIK3CD* mutant. (E, F) Flow cytometry analysis of naïve (CD45RA^+^CCR7^+^), TCM (CD45RA^−^CCR7^+^), TEM (CD45RA^−^CCR7^−^), effector (CD45RA^+^CCR7^−^) T cells in PBMCs from CD3^+^CD4^+^ (E) and CD3^+^CD8^+^ (F) T cells of the mother with *PIK3CD* mutant. Shown are representative dot plots. (G, I) Statistics of percentage (±SEM) of naïve (CD45RA^+^CD27^+^), TCM (CD45RA^−^CD27^+^), TEM (CD45RA^−^CD27^−^), effector (CD45RA^+^CD27^−^) T cells in CD3^+^CD4^+^ (G) and CD3^+^CD8^+^ (I) T cells from HCs and the mother with *PIK3CD* mutant. (H, J) Statistics of percentage (±SEM) of naïve (CD45RA^+^CCR7^+^), TCM (CD45RA^−^CCR7^+^), TEM(CD45RA^−^CCR7^−^), effector (CD45RA^+^CCR7^−^) T cells in CD3^+^CD4^+^ (H) and CD3^+^CD8^+^ (J) T cells from HCs and the mother with *PIK3CD* mutant.

**Figure S2. *PIK3CD* mutation leads to over-activated B cells and PI3K/AKT/mTOR signaling pathways with FOXO1 binding to *CD38* promotor.**

(A, B) PBMCs from HCs and the mother were pre-incubated with anti-CD19, followed by stimulation with Biotin-SP-AffiniPure F(ab')_2_ Fragment Goat Anti-Human IgG + IgM (H+L) for 0 and 15 min. After fixation and permeabilization, cells were stained with anti-pPI3K, anti-pFOXO1, anti-pAKT, anti-pmTOR, anti-pS6 and analyzed by flow cytometry. The MFI of pPI3K, pFOXO1, pAKT, pmTOR, pS6 in CD19^+^ B cells were quantified by FlowJo 10 software.
